# Barriers to medication adherence for the secondary prevention of stroke: a qualitative interview study in primary care

**DOI:** 10.3399/bjgp16X685609

**Published:** 2016-05-24

**Authors:** James Jamison, Jonathan Graffy, Ricky Mullis, Jonathan Mant, Stephen Sutton

**Affiliations:** Professor of behavioural science, Behavioural Science Group;; Primary Care Unit, Institute of Public Health, University of Cambridge School of Clinical Medicine, Cambridge.; Primary Care Unit, University of Cambridge, Strangeways Research Laboratory, Cambridge.; Primary Care Unit, University of Cambridge, Strangeways Research Laboratory, Cambridge.; Professor of behavioural science, Behavioural Science Group;

**Keywords:** general practice, medication adherence, qualitative research, secondary prevention, stroke

## Abstract

**Background:**

Medications are highly effective at reducing risk of recurrent stroke, but success is influenced by adherence to treatment. Among survivors of stroke and transient ischaemic attack (TIA), adherence to medication is known to be suboptimal.

**Aim:**

To identify and report barriers to medication adherence for the secondary prevention of stroke/TIA.

**Design and setting:**

A qualitative interview study was conducted within general practice surgeries in the East of England, UK.

**Method:**

Patients were approached by letter and invited to take part in a qualitative research study. Semi-structured interviews were undertaken with survivors of stroke, caregivers, and GPs to explore their perspectives and views around secondary prevention and perceived barriers to medication adherence. Key themes were identified using a grounded theory approach. Verbatim quotes describing the themes are presented here.

**Results:**

In total, 28 survivors of stroke, including 14 accompanying caregivers and five GPs, were interviewed. Two key themes were identified. Patient level barriers included ability to self-care, the importance people attach to a stroke event, and knowledge of stroke and medication. Medication level barriers included beliefs about medication and beliefs about how pills work, medication routines, changing medications, and regimen complexity and burden of treatment.

**Conclusion:**

Patients who have had a stroke are faced with multiple barriers to taking secondary prevention medications in UK general practice. This research suggests that a collaborative approach between caregivers, survivors, and healthcare professionals is needed to address these barriers and facilitate medication-taking behaviour.

## INTRODUCTION

Stroke is responsible for around 11% of all deaths worldwide.^1^ Approximately 17 million incidences of first-time stroke occurred in 2010.^2^ Every year, stroke causes in the region of 5.7 million deaths.^3^

Reducing the stroke burden and risk of further cerebrovascular events can be achieved through implementing cholesterol lowering and blood pressure lowering therapies.^4^^–^6 However, prevention is dependent on the survivors adherence to medication. Estimates suggest around 50% of patients with chronic disease are nonadherent,^7^ resulting in significant adverse outcomes as well as increased morbidity and mortality.^8^

Medication adherence is suboptimal among survivors of stroke. A systematic review exploring whether adherence to cardiovascular therapies influenced the risk of cardiovascular disease (CVD) concluded that a significant proportion of people did not adhere to cardiovascular medications and as much as 9% of all CVD events in Europe could be attributed to poor adherence to vascular medications alone.^9^ Elsewhere, De Simoni and colleagues found few trial interventions supporting the effect of medication adherence on lowering blood pressure in survivors of stroke or transient ischaemic attack (TIA). Trials, which included highly-selected stroke populations, largely excluded patients with any significant cognitive deficit and did not account for the caregiver role in the lives of stroke patients.^10^

There have been few studies on adherence barriers in stroke. Kronish and colleagues identified concerns about medication and knowledge of stroke prevention therapies as important barriers among survivors of stroke.^11^ In another study, beliefs about medication, side effects of medication and the inadequate provision of information were considered important barriers to medication adherence.^12^ Further evidence on factors affecting adherence after stroke could address the poor uptake of these medications. The aim of this study was to use qualitative interviews to explore the barriers to medication adherence in UK general practice.

## METHOD

### Design and participants

Interviews were conducted with patients on the stroke registers of five GP surgeries, together with their carers where relevant, and one GP from each practice. A list of patients aged >55 years, with a history of stroke or/TIA was compiled and sent to the GPs for review. Anyone who was considered unfit to participate in the research (that is, was seriously ill or terminally ill) was excluded and not approached by the practice. To achieve a maximum spread of age, socioeconomic status (Indices of Multiple Deprivation score^13^), sex and disability (using modified Rankin score^14^), purposive sampling was undertaken. Initially, 25 patients from each practice were approached by letter. Positive responders were telephoned to confirm their attendance and the presence of a caregiver at the interview. The final number of interviews was determined by the point of data saturation.

**How this fits in**

Medication nonadherence is known to be problematic among stroke survivors, contributing to suboptimal health outcomes. Multiple barriers to medication adherence have been identified and characterised as specific to the individual, the medication, or the healthcare setting. This qualitative investigation of the attitudes of 28 survivors, 14 caregivers, and five GPs found patient barriers, such as their knowledge of stroke and medication, and medication barriers, such as patient beliefs about treatment, to be important. A collaborative approach is needed to address important barriers to medication adherence in UK general practice.

### Semi-structured interviews

Semi-structured interviews provided an opportunity for in-depth investigation of people’s personal perspectives, using an open-ended line of questioning, which defined the area to be explored.^15^ A topic schedule guided the line of questioning and prompts encouraged further discussion (Appendix 1). Two survivors piloted the patient topic guides and recommendations were incorporated. A clinical researcher provided feedback on the GP topic guide. Interviews were conducted in the patient’s home or the practice. Discussion topics included attitudes to secondary prevention care, medication beliefs, adherence to treatment, carer role, GPs attitudes towards current practice, and barriers to uptake. Interviews were conducted between June 2013 and February 2014, lasted 1.0–1.5 hours and were audiotaped and transcribed.

### Data analysis

To ensure reliability of interpretation, transcripts were initially read by one of the authors and inaccuracies resolved by listening to the recordings. NVivo 9 was used to organise, code, and manage the data. Transcripts were entered into the program and coded, using grounded theory and followed a constant comparative analysis^16^ approach, in which key points were identified from the data and coded individually. An iterative process of data collection and data analysis was undertaken. Initially, chunks of data were coded. Codes were then grouped into similar concepts and themes and categories were formed. A process of identification and refinement of categories followed. As groups were compared further, more abstract categories developed until the core themes emerged. To strengthen the validity of findings and ensure rigour, 20% of all interviews were double-coded by a second member of the research team. Inconsistencies were resolved through discussion with a third author until a consensus on the final themes was reached.

## RESULTS

In total, 33 interviews were completed: five with GPs and 28 with stroke survivors, 14 of whom had a caregiver present. The characteristics of the stroke survivors are presented in Appendix 2.

**Figure 1.**
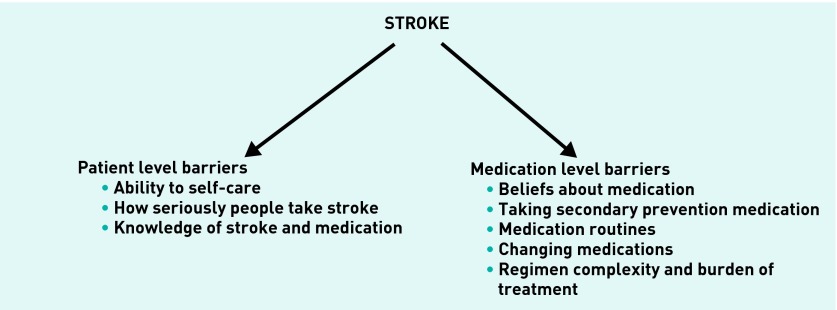
***Barriers to medication adherence among survivors of stroke/transient ischaemic attack.***

Two key themes were identified. The first theme was patient level barriers and this included the subthemes ability to self-care, how seriously people take stroke, and knowledge of stroke and medication. The second main theme was medication level barriers and this included subthemes beliefs about medication, taking secondary prevention medication, medication routines, changing medications, and regimen complexity and burden of treatment. Figure 1 shows the key themes and subthemes identified.

### Patient level barriers

#### Ability to self-care

GPs admitted that being housebound was a significant barrier compromising patients care and affecting adherence:

‘If somebody is stuck at home, a total 5 hours they have got contact with somebody, the rest of the 365 days they are by themselves … their outcome is likely to be worse … their care can be low. If they’re depressed they won’t take their tablets.’

(GP05, male [M])

For many survivors, meanwhile, dependence on a caregiver for their knowledge and managing medication was important:

*‘My wife sorts it out and that’s why I don’t know so much about it you see she* [taps]. *She puts them there, I take them and that’s it.’*

(Patient [P] 04, M, 80 years)

#### How seriously people take stroke

Survivors and carers frequently trivialised stroke and the significance of symptoms, often due to a lack of knowledge and expectation around the condition:

‘I knew there was a problem but I thought perhaps it would go away. So you sort of erm bury your head in the sand.’

(P20, female [F], carer)

‘Within sort of half hour, hour at the most I felt I was ok again. The fact that we drove home the next day without seeking medical attention, it’s silly but I did it because I didn’t think anything else about it, it’s gone whatever it is.’

(P09, M, 68 years).

‘I wouldn’t take them because I still, to me, blood pressure and cholesterol tablets to me, I don’t see what they’re doing for me.’

(P24, M, 75 years).

In the absence of symptoms, the need for medication was also frequently underestimated, as GPs confirmed:

‘If they don’t see it or it didn’t leave any residual effect on them, then they tend to forget these things. Out of the sight, out of the mind.’

(GP02, F)

#### Knowledge of stroke and medication

Inadequate information on stroke prevention and recovery was frequently cited by patients and caregivers:

‘No, I don’t think we’ve got hardly any information. We haven’t ever really had a lot of information about it have we? You just sort of get on with it … I mean perhaps I haven’t ever asked enough but … I think you should be, told in advance.’

(P14, M, carer)

In addition, level of knowledge varied with several survivors admitting to being well-informed, while others felt confused about tablets and the reasons they needed to take them:

‘The importance of taking these exactly on time is trivial. I would probably survive for a week, if I didn’t take them. For a month I’d probably survive. It would not make any difference in 2 days.’

(P03, M, 86 years)

‘I don’t know why I take them but it tells you on each one you know what it’s for … I wouldn’t say I know what they’re for.’

(P16, F, 82 years)

GPs agreed survivors lacked knowledge of medications but that many took tablets just because the doctor told them to:

‘I would say 50% of patients know what medication they are taking but erm 50% of patients doesn’t know, they think the doctors have prescribed me this medication and I have to take it and that’s why they are taking it.’

(GP02, F)

### Medication level barriers

#### Beliefs about medication

Patients’ beliefs about medication frequently dictated adherence to some drugs:

‘I think aspirins are good for you. That’s the only one I fancy. Well it thins the blood and I think, well by thinning the blood it flows better and that stops any clots so I do like to take it. I just don’t see why I’m taking other medication, I’m not fat or anything like that. I don’t get very high blood pressure and well cholesterol, what is cholesterol?’

(P24, M, 75 years)

‘*I refused it and … I said well … it’s not because it’s rat poisoning. If you tell me I’ve got warfarin I must be ill and if I take aspirin I can’t be that ill*.’

(P22, F, 71 years)

Some survivors questioned the need for any medication, expressing doubts despite experiencing a stroke:

‘I mean I’m taking them because they know better than I do, but at the same time at the back of my head I’m thinking I, I shouldn’t have to take those.’

(P10, M, 66 years)

Some participants focused on conditions with a greater impact on everyday health:

‘To me the most important thing for her is controlling her diabetes … because I don’t want her passing out having a diabetes wobbly.’

(P08, M, 87 years)

#### Taking secondary prevention medication

The importance of taking stroke medication was widely acknowledged, however, total adherence was a minor concern for most:

‘I’m sort of, a little bit annoyed that I’ve missed them but, no it doesn’t worry me. It would worry me if … I missed them for 3 or 4 days but a day, no.’

(P10, M, 66 years)

Although most patients considered themselves adherent, many reported forgetting to take their night medication:

‘Well now and again I forget the cholesterol because that’s the one at night and it’s the only one I take at night.’

(P15, M, 67 years)

For some survivors, not taking medication was a conscious decision and GPs acknowledged they needed to respect this:

‘We do have to respect their autonomy at the end of the day it’s their bodies and some of them say to me look, for goodness sake I’m 94, I don’t want to take these tablets, it makes me feel ill. I do have to respect that.’

(GP01, F)

Nevertheless, survivors and caregivers reported they were generally happy to follow the advice of their GP:

‘So if the doctor says take ten pills a day, I’ll, I’ll do it … he makes the decision and erm he, he’s the boss man as you might say, who knows what he’s up to.’

(P08, M, 87 years)

Patients also identified practical barriers including difficulties accessing medications and the size of tablets:

‘The big ones, I, do actually feel I have to swallow two or three times to get them down.’

(P10, M, 66 years)

‘Some of the, the pills are a hell of a trouble, you know the bubble wrap, flipping them out especially with my hands not as strong as they should be.’

(P08, M, 87 years)

#### Medication routines

Many patients admitted following a medication-taking routine, without which they would have difficulties with medication adherence:

‘I only remember to take the others be … if I take them out of the cupboard the night before and leave them on the top. If I didn’t take them out, I, I, would probably forget … because it isn’t the first thing that I think of … you know when I, when I first get up.’

(P10, M, 66 years)

The use of medication blister boxes was also beneficial and improved the experience of taking tablets:

*‘* [Taking medication] *that was a lot more hit and miss then … when you pop ‘em open if one flies on the floor I think, nah leave it. Sweep it up later on. It’s like a pleasure doing it now.’*

(P06, M, 61 years)

#### Changing medications

Survivors of stroke described how medications were frequently changed, leading to disruption in pill administration and unwanted treatment side effects:

‘I did have a bad run because they changed the looks of the tablets oh god and I was taking four gout tablets a day and no diabetes ones and that put the old sugar up.’

(P13, M, 70 years)

*‘They changed his medication to cheaper cholesterol and* [he] *was physically ill. He couldn’t cope on it at all so he went back and the doctor said “oh well it was just to try” and they put him back on the others.’*

(P24, F, carer)

#### Regimen complexity and the burden of treatment

Survivors frequently expressed concerns around the burden of treatment with several describing how visiting the GP often resulted in additional medications:

*‘I have to take 10 a day now altogether but I went up there* [to the practice] *to say can I get off some of these tablets, and I come back and I was on an extra one so I’ve not been up since.’*

(P13, M, 70 years)

Others felt that the increased burden only contributed to their lack of understanding around stroke medications:

‘I’ve got yards of them. I don’t know half the names I’m just told when to take them. That’s one thing I’d like to do away with.’

(P11, M, 73 years)

GPs also acknowledged the burden of treatment and the contribution to patient’s negative attitudes towards taking medication:

‘Most of them are more unhappy about the number of tablets … from a patient’s perspective it’s usually it’s just physically a lot of tablets you have to swallow.’

(GP03)

Among the older patients, increased burden often led to a choice being made between which medications to take:

‘Seventy per cent of patients are fully compliant but some of them are not compliant with these medications especially the elderly group of the patients because they think they are taking too many medications and so … they keep missing out the medications.’

(GP02, F)

## DISCUSSION

### Summary

A qualitative interview study was conducted, with survivors of stroke, caregivers, and GPs, to explore barriers to medication adherence in the UK general practice setting. Two key themes were identified. The first, patient level barriers, included the subthemes ability to self-care, knowledge of stroke and medication, and survivor’s tendency to trivialise stroke. The second key theme, medication level barriers, included the subthemes beliefs about how pills work, importance of taking medication, attitudes to missing tablets, difficulties taking medications, changing medication, and burden of treatment.

### Strengths and limitations

An important strength was the inclusion of caregivers and GPs alongside patients, providing diversity of opinion. Employing a semi-structured interview methodology allowed participants maximum scope to dictate the direction of conversation and permitted an in-depth assessment of the topic area. This study offers a unique perspective on medication adherence barriers through the perceptions of stroke survivors, caregivers, and GPs. Due to the small number of GPs, it is unclear whether these views are representative of health professionals. Recruitment through five practices may have also limited the potential to generalise findings to the wider stroke population. Survivors were predominantly white, few were significantly disabled, and none had substantial cognitive impairment. Future research could include patients with aphasia who are dependent on others and patients from ethnic minorities in whom CVD is known to be prevalent.

### Comparison with existing literature

Similar investigations from France and UK also reported that lack of symptoms and knowledge were important barriers to adherence.^12^^,^^17^ Poor knowledge contributed to misunderstanding, with stroke frequently trivialised and its symptoms ignored. This is perhaps not surprising, given that half of this study’s sample reported experiencing a TIA or mini-stroke, where symptoms usually disappear within 24 hours. Indeed, this absence of symptoms has often been identified as an important reason for the lack of urgency among survivors seeking help following stroke onset.^18^ Elsewhere, a systematic review of qualitative studies on patients’ understanding of hypertension and medication-taking identified side effects and a dislike of medication as key reasons for not continuing treatment.^19^

Lack of knowledge, doubts about treatment efficacy and prioritising medications are in line with previous work in which poor adherence was linked with being likely to question the purpose of medication, having a poor understanding of therapy and concerns around the lack of information provided by the health professional.^20^ Prioritising medications due to perceived importance and treating the most salient symptoms corresponds with patients performing a risk–benefit assessment, in which condition severity and knowledge of medication influence the decision to use treatment.^21^ The potential for positive beliefs on medication to influence subsequent behaviour suggest that exploring beliefs among survivors of stroke should be considered in an effort to improve medication adherence.^22^ The lack of knowledge identified among stroke survivors and caregivers suggests a need for improved education around stroke and treatment of the condition. Although education is a key component of providing stroke care, both survivors and caregivers face considerable barriers to information.^23^

The present investigation confirms previously reported barriers, including difficulties swallowing or accessing medication;^8^ frequent changing of medication;^24^ use of storage devices;^25^ treatment complexity; and the influence of comorbidities.^26^ Complex medication regimens are important factors in adherence to chronic conditions, including hypertension and CVD.^27^ While reducing the daily medication dose can improve adherence to antihypertensives,^28^ recent research has suggested a fixed-dose combination (FDC) polypill can improve adherence to medications^29^ and address the barriers reported here.

These findings add to the growing body of literature on barriers to medication adherence in stroke. The failure of patients to act on stroke symptoms may represent a broader lack of knowledge associated with experiencing a TIA. Research into behaviour following a TIA indicates that a delay in seeking treatment is not uncommon, attributed not only to the recognition of symptoms but also the role of others and interactions with the healthcare provider.^30^ This study highlights the important role of the caregiver in providing information and facilitating medication-taking behaviour. Further work exploring the role of the caregiver is therefore warranted. Inadequate stroke knowledge and information provided by the GP has been reported previously, indicating there are significant unmet needs within this group.^31^ While the measurement of adherence was beyond the scope of the current study, exploring how the beliefs and perspectives of survivors reflect actual levels of adherence should also be considered. Determining how the barriers identified here relate to actual adherence may help determine where secondary prevention efforts should be focused in the future.

### Implications for practice

These findings provide an important basis from where effective adherence interventions to improve stroke care may be developed and implemented in clinical practice. Interventions are needed to address barriers to medication adherence among survivors of stroke and ultimately improve stroke outcomes within this population. This study suggests that increased efforts to improve awareness of stroke and secondary prevention medication is warranted. Given their potentially significant role in managing medication, it is important that caregivers are fully engaged with efforts aimed at addressing barriers and improving adherence to stroke medication. It is likely that caregiver support may be important for maintaining adherence among those survivors with cognitive limitations, who were largely overlooked in the present study, and who may themselves face considerable barriers to adherence.

Adopting a collaborative approach between the patient, caregiver, and practitioner, as well as the wider primary healthcare team of practice nurses and pharmacists, who can also play a role in facilitating adherence, should be considered and can be a focus for future work in this area. Finally, developing the patient–practitioner relationship and facilitating better communication can enhance survivors’ understanding and knowledge of stroke and medication, while encouraging better adherence through challenging barriers to treatment.

In conclusion, important barriers to stroke medication adherence within UK general practice have been identified. Interventions are needed to address challenges associated with suboptimal adherence, including the provision of inadequate information, the role of the caregiver, recognition of stroke symptoms, patient beliefs about medication, and the burden of secondary prevention treatment. This investigation provides insight on the perspectives of practitioners, caregivers, and survivors, highlighting the complex and multifactorial barriers they face to stroke medication.

## Appendix 1. Interview schedule

**Table:**

**Patient**

Can you tell me about your health since you had your stroke? How would you say your health has changed? In what way, if any, has the stroke changed your relationship with your carer? Is there anything you find particularly difficult since you had your stroke? Can you tell me about the stroke medication you currently take? What are your general feelings towards taking your current stroke medication? Do you know understand what the medications you take are for? How is your medication managed? Who is responsible? How does this work? Would you like to manage your own medication? Do you think this is an important role? Taking your medication: Do you always take your medication when you’re supposed to? Do you experience any other problems taking medication? Can you tell me a bit more about these problems? (for example, is quantity/size of meds a problem, etcetera). In what ways do you think the process of taking your medication could be improved?

**Caregiver**
Can you tell me about your experiences as a caregiver? What is this like day-to-day? How has this changed your relationship with the patient? What would you say has been most difficult about this experience of being a carer? Do you manage the patient’s medication? If so, can you tell me a little bit about this role? Have you always managed their medication? If not, why? How important would you consider the role of managing this medication? How good is the patient at taking his/her medication? Are there any difficulties *around the* taking of stroke medication. What do you think is the main concern the *patient* has? (for example, size, quantity, forgetting). How do you think the medication-taking process could be made easier/improved?

**GP**
What do you think of current treatment for secondary prevention of stroke? How do you think current stroke treatment/medication regimens could be improved? Do you think current regimens are easy for patients to understand/manage? Can you think of any limitations of current regimens for secondary prevention?

## Appendix 2. Interview participant characteristics

**Table:**

**ID number**	**Age, years**	**Sex**	**Time since stroke/TIA, years**	**Stroke classification**	**Diabetic status**	**Smoking status**
P01	71	Female	18	Stroke	Not diabetic	Non-smoker
P02	65	Male	3	Stroke	Diabetic	Smoker
P03	86	Male	5	Stroke	Not diabetic	Non-smoker
P04	80	Male	2	TIA	Not diabetic	Ex-smoker
P05	64	Male	10	TIA	Not diabetic	Non-smoker
P06	61	Male	7 months	TIA	Diabetic	Smoker
P07	67	Male	4.5	Stroke	Not diabetic	Non-smoker
P08	87	Male	2	TIA	Diabetic	Non-smoker
P09	68	Male	3	TIA	Not diabetic	Ex-smoker
P10	66	Male	3	TIA	Not diabetic	Ex-smoker
P11	73	Male	13	Stroke	Not diabetic	Ex-smoker
P12	67	Male	2	Stroke	Diabetic	Ex-smoker
P13	70	Male	10	TIA	Diabetic	Ex-smoker
P14	66	Female	6	Stroke	Not diabetic	Non-smoker
P15	67	Male	6 months	Stroke	Not diabetic	Ex-smoker
P16	82	Female	4 months	TIA	Diabetic	Non-smoker
P17	79	Male	10	TIA	Not diabetic	Non-smoker
P18	88	Male	12	Stroke	Diabetic	Non-smoker
P19	93	Male	10	TIA	Not diabetic	Non-smoker
P20	89	Male	2	TIA	Not diabetic	Non-smoker
P21	68	Female	3.5	Stroke	Not diabetic	Ex-smoker
P22	71	Female	9	TIA	Not diabetic	Non-smoker
P23	74	Female	5	TIA	Not diabetic	Non-smoker
P24	75	Male	2	Stroke	Not diabetic	Non-smoker
P25	76	Male	9 months	Stroke	Diabetic	Ex-smoker
P26	72	Male	17	TIA	Diabetic	Non-smoker
P27	73	Male	2	Stroke	Not diabetic	Ex-smoker
P28	74	Female	4	Stroke	Not diabetic	Ex-smoker
